# Perceptions and practices of spiritual care among hospice physicians and nurses in a Taiwanese tertiary hospital: a qualitative study

**DOI:** 10.1186/s12904-020-00608-y

**Published:** 2020-07-01

**Authors:** Zoe Tao, Poshu Wu, Amber Luo, Tzu-Lin Ho, Ching-Yu Chen, Shao-Yi Cheng

**Affiliations:** 1 School of Medicine, The University of Texas Southwestern Medical Center, Dallas, TX USA; 2 Department of Palliative Medicine, Koo Foundation Sun Yat-Sen Cancer Center, Taipei, Taiwan; 3 Department of Applied Psychology, New York University, New York, NY USA; 4 Department of Family Medicine, College of Medicine and Hospital, National Taiwan University, No.1 Chang-de Street, Zhongzheng District, Taipei, Taiwan 10048

**Keywords:** Cancer, Oncology, Hospice, Spirituality, Psychosocial care, Asia

## Abstract

**Background:**

Spiritual care is frequently cited as a key component of hospice care in Taiwanese healthcare and beyond. The aim of this research is to gauge physicians and nurses’ self-reported perspectives and clinical practices on the roles of their professions in addressing spiritual care in an inpatient palliative care unit in a tertiary hospital with Buddhist origins.

**Methods:**

We performed semi-structured interviews with physicians and nurses working in hospice care over a year on their self-reported experiences in inpatient spiritual care. We utilized a directed approach to qualitative content analysis to identify themes emerging from interviews.

**Results:**

Most participants identified as neither spiritual nor religious. Themes in defining spiritual care, spiritual distress, and spiritual care challenges included understanding patient values and beliefs, fear of the afterlife and repercussions of poor family relationships, difficulties in communication, the patient’s medical state, and a perceived lack of preparedness and time to deliver spiritual care.

**Conclusions:**

Our study suggests that Taiwanese physicians and nurses overall find spiritual care difficult to define in practice and base perceptions and practices of spiritual care largely on patient’s emotional and physical needs. Spiritual care is also burdened logistically by difficulties in navigating family and cultural dynamics, such as speaking openly about death. More research on spiritual care in Taiwan is needed to define the appropriate training, practice, and associated challenges in provision of spiritual care.

## Background

Spiritual care remains an integral component of Taiwanese hospice care, with indigenous spiritual care taking root in the first tertiary inpatient palliative care unit in Taiwan by employing Buddhist chaplains to care for the patient in death preparation [1]. Currently it is unclear how Western definitions of spiritual care practices, such as aiming to improve patient quality of life and coping with symptoms, may hold in the context of Taiwanese hospice care [2, 3]. The objective of our study was to synthesize participant perspectives through semi-structured interviews to ascertain what it means to be a provider of spiritual care in a Taiwanese urban hospice setting. We sought to gauge physicians and nurses’ self-reported perspectives and current practices on addressing spiritual care.

### Spiritual care in Taiwan

How do health professionals define spiritual care as a discrete clinical phenomenon? Spiritual care has been distinguished from religious care in its non-specificity to a faith tradition, but may still hold religious influences [1, 4]. Provision of spiritual care has also been known to influence medical treatment intensity at the end of life [5]. A systematic review on discussing spirituality in a medical setting demonstrated that patients desire to discuss spiritual concerns, but medical teams infrequently provide spiritual care; however, life-threatening illness can make a physician more likely to provide spiritual care [6]. An international survey encompassing nearly 300 palliative care physicians across 87 countries demonstrated that support for researching spiritual care was high on a global scale to guide best practices in palliative care [7].

Spiritual care has received increased attention in Taiwanese inpatient settings in recent years, with the first private hospice in Taiwan founded with a Christian orientation and the first public hospice at National Taiwan University Hospital with a Mahayana Buddhist orientation in the 1990’s [1, 8]. Clinical Buddhist Chaplains (CBC’s) remain a mainstay of Taiwanese hospice care, and often provide spiritual guidance to patients and families during life-limiting illness [1, 9]. Spiritual care is conducted by CBC’s through identifying patient’s and family’s physical, psychological, and spiritual suffering and planning care according to traditional Buddhist dharma teachings, effectively reducing fears surrounding the dying process [9]. Of note, while the term “spiritual care” in Mandarin Chinese may hold an additional connotation of death acceptance and subsequent peace of mind, the term itself serves a similar function in Chinese as it does in English for purposes of hospital care [10]. Outside of chaplaincy, the role of spiritual care in Taiwanese healthcare practice is less defined [11]. Hospice and palliative care physicians in the National Taiwan University Hospital are primarily family medicine physicians, who receive mandatory hospice training in Taiwan; spiritual care, including learning the role of CBC’s, is a mandatory topic in their training curriculum [12]. For nurses working in hospice, more initiatives in spiritual care training and research have been undertaken across Taiwanese institutions [8, 13]. As all palliative physicians and nurses in Taiwanese healthcare are also hospice staff, hospice and palliative will be used interchangeably to refer to our physician and nurse populations [12].

In Taiwan, caring for patients with advanced illness has been influenced by sociocultural taboos surrounding speaking the truth of adverse health circumstances openly [14–16]. A phenomenon describing families performing medical decisions on behalf of the patient to mitigate their spiritual and psychosocial distress can prevent candid discussion of terminal illness; however, it is unclear how such sociocultural phenomena may specifically impact spiritual care practices among healthcare providers [16]. Taiwanese family practice physicians and nurses, for whom hospice and palliative care training is mandatory, are the mainstay healthcare providers in Taiwanese hospice settings [12]. A 2019 task force representing six Asian countries on advance care planning stated the spiritual domain was essential to end of life care; the same expert panel provides a reminder that Asian people generally regard death as a cultural taboo to discuss openly and urges providers to perform advance care planning discussions with sensitivity to said taboo [17]. Based on Taiwanese end of life care literature suggesting 1) great interest in spiritual themes in inpatient care and 2) difficulties in candid discussion of death and dying due to sociocultural norms, interviewing physicians and nurses about their experiences in spiritual care can yield valuable information about how spiritual care is perceived and practiced in the inpatient hospice setting and what challenges healthcare staff may face by involving themselves in spiritual care [15–18].

## Methods

We recruited a total of 20 participants through purposive sampling during weekday working hours within the palliative care unit physician and nurse working area of a single tertiary care hospital (Table 1). We excluded those with uncompensated work in the hospice, non-familiarity with Mandarin Chinese, and inability to provide informed consent. We invited 23 total participants and 3 refused, citing conflicting obligations. We identified physicians and nurses who had worked in hospice care for at least 1 year at National Taiwan University Hospital (NTUH); we excluded those participating as study personnel for concerns of biasing interviews. IRB approval was granted from National Taiwan University Hospital [IRB number 201805065RINC, approved 6/8/18], and all individuals signed a written consent form before participation.

**Table 1:** Demographics of study participants

Characteristic	
Years Working in Palliative Care	
1–5	7 (35%)
6–10	6 (30%)
11–15	3 (15%)
16–20	2 (10%)
21+	2 (10%)
Profession	
Physician	9 (45%)
Nurse	11 (55%)
Religion	
Religious/Spiritual	7 (35%)
Non-religious/Non-spiritual	13 (65%)
Gender	
Female	14 (70%)
Male	6 (30%)

**Fig. 1:**
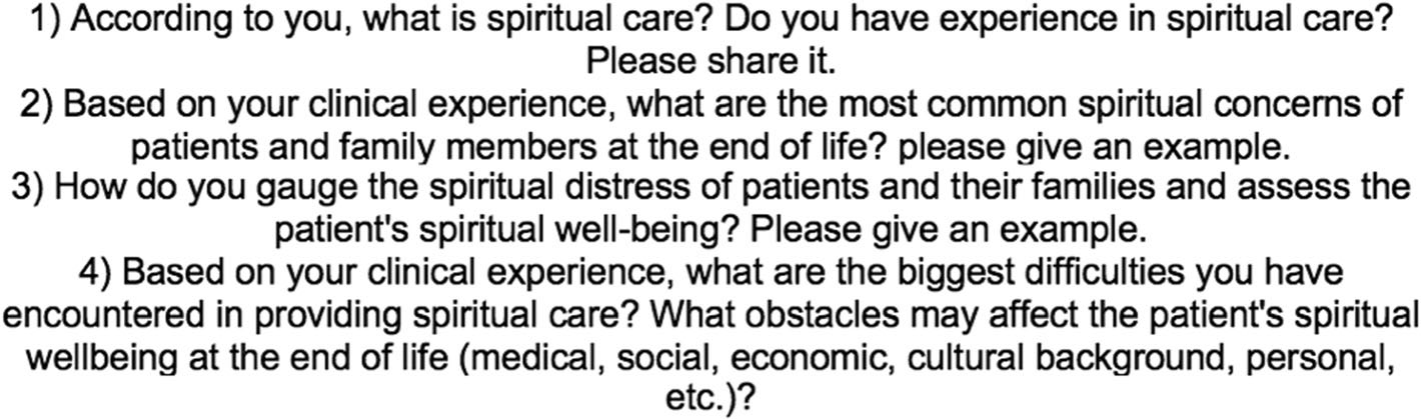
Interview outline provided to participants (translated to English)

Interviews were conducted in July 2018 by ZT, a medical student with a degree in Religion and Psychology with prior experience in qualitative research unaffiliated with NTUH outside of the study. Prior to initiation of interviews, she was observed and given feedback by the supervising faculty members of the study practicing and recording 3 mock interviews with family medicine residents who had worked in the hospice ward. Demographic information was obtained from participants immediately prior to the interview. Interviews lasted an average of 20 min and were semi-structured in nature, where participants responded in an open-ended manner to a series of four set questions with encouragement to elaborate if applicable and verbal probes to share experiences [19]. Interviewees were given a written copy of the questions as a guide (Fig. 1). Interviews were conducted in Mandarin Chinese and recorded using a portable recording device. The attending physician members of the study did not have access to interview transcripts or recordings for house staff confidentiality. Interviews were transcribed in Chinese using dictation software, proofread and edited by a staff research assistant, then translated into English by the second student assistant. The interviewer was present at every part of the transcription and translation process and was available to answer questions and resolve discrepancies where present.

### Data analysis

We employed a qualitative design with semi-structured interviews and a directed approach to content analysis of the interviews. Directed content analysis is an approach in qualitative methodology which validates or expands on an existing theoretical framework or theory and differs from conventional content analysis in its usage of pre-determined broad categories for analysis [19]. Interview content was organized using Corpus Tool software. We employed prolonged engagement (ZT), peer review, clarifying researcher bias, a second coder, and development of a coding system derived from the interview questions (Fig. 1) for data reliability and validity [20].

The second round of coding involved a summative process tallying the number of participants for which individual themes appeared from these three categories. Overall, both theme frequency and qualitative emphasis, determined by group consensus, were utilized to select emerging themes for presentation. A primary coder completed the first review of interview content by reading interview transcripts in English, taking notes, and developing themes such as “communication” and “pain”; lines of interviews coded as multiple themes or subthemes were resolved by group consensus. At this time, the number of individual interviews in which each subcategory appeared throughout the coded portions of the interviews was noted, along with exemplar quotes pertinent to the theme. Themes were then selected for analysis based on their frequency of appearance and qualitative emphasis, which was determined by consensus of three members of the team in a single meeting. Multiple themes were compiled into one if they had significant content or quotation overlap, also determined by group consensus. TH reviewed transcripts and themes at each stage of the coding process independently to ensure integrity of the derived themes with interviews.

## Results

The study participants are described in Table 1. Out of 20 participants, 7 identified as “spiritual,” with 3 of these participants identifying as Buddhist, 1 as Christian, and 3 as spiritual but not religious. The amount of time working in hospice care ranged from 1 to 23 years. All nurses were female and out of the physicians, 6 were male and 3 were female. 9 participants were physicians and 11 were nurses. Results are organized by responses to individual questions (broad categories): definitions of spiritual care, sources of spiritual distress, and challenges in providing spiritual care. Examples are shown both in Table 2 and below.

**Table 2:** Interview categories, themes, and quotations

***Category***	***Themes and Quotations***
*1. Definitions of spiritual care.*	Patient beliefs and values “You have to go get to know a person. What is his/her value in life? What is his faith? Or are there things that he is afraid of?”
	Providing presence “A lot of patients see that your care is always there, no matter what state he’s in. if he feels this power of warmth, he can ride the wave of this strength.”
	Addressing physical symptoms refractory to medical management “The first thing would be when there are a lot of physical symptoms and after looking at test results or blood results, he shouldn’t be that uncomfortable. However, he expresses discomfort a lot with a lot of symptoms. This hints to me as spiritual troubles.”
	Addressing psychological or emotional needs “To me, it’s very difficult to differentiate between spirituality and psychology…I think it’s a psychological and emotional thing, and towards walking to this stage of life, where are we going then?”
*2. Sources of spiritual distress.*	The afterlife “Some Buddhists may worry that they won’t be able to go to a better world after they pass away and may go to hell. Family members would also worry about this sometimes.”
	Burdening one’s family “Some people may think that when they were young, they were irresponsible to their families, so their children are not visiting them now.”
	Communication “…because we always discuss the issue of death very vaguely and discreetly, a lot of people don’t know that they are in the final stage of life. This type of uncertainty makes them not know how to react and adjust to themselves losing bodily functions, and don’t know how to do the following preparations.”
*3. Challenges in providing spiritual care.*	The state of the patient “…when the symptoms worsen, the physical discomfort would affect the practicality of him [chanting prayers]. It’d end up becoming that he isn’t able to change anymore…these physical symptoms would affect the feasibility of us performing spiritual care.”
	Providers’ lack of preparedness *“*I think the biggest problem is still the inability to notice, or sometimes when you’re working, you may not be mature enough to explore this area…or the worries of noticing things that you’re unable to help with.”
	Providers’ lack of time “Everybody’s too busy.”

### Definitions of spiritual care

A recurrent theme in response to the question of defining spiritual care was understanding patient values and beliefs, but responses varied greatly. They included how the patient sees the meaning of life, figuring out the role of a patient’s faith in their medical circumstances, and providing a caring presence to patients. Of note, many providers expressed “this is hard” or “this is pretty difficult to define” prior to giving a definitive answer to this first question. Participants frequently made mention of the relationships between psychological, physical, and spiritual phenomena. Several reported the assessment of physical symptoms, especially ones refractory to medical management, as spiritual care, and others focused exclusively on meaning and faith.

“So I usually, for example, would review his life experiences with him, for example, work experiences, or some experiences with his family, and through that figure out what this person’s values were and are.”

“So mainly we make sure that physical problems have been addressed, then we can help with their spiritual and psychological states.”

“I think spiritual care has two levels: one is belief, one is faith…belief refers to for example what he thinks the meaning of life is, and faith refers to the traditional religions.”

“We look at whether or not his physical comfort level can be properly controlled by the adjustments of medication.”

### Sources of spiritual distress

A frequently reported source of spiritual distress was the idea of the afterlife, especially whether one would still be in pain after death. This was often mentioned with the theme of poor family relationships.

“In clinical settings, we aren’t able to tell patients where they’ll go [after death] for sure. We can only listen to them, reassure them of all the good things they’ve done in their life that they really did them well.”

“A lot of patients would think that they won’t get better, they’re just dragging the time out, just spending money, spending family’s money, spending family’s time and energy, and becoming a really heavy burden.”

“Some people may think that when they were young, they were irresponsible to their families, so their children are not visiting them now.”

“The most difficult things are probably relationships…his family may be willing to take care of the post-death issues, but aren’t willing to take care of him. So that patient didn’t really talk much.”

“At the time, the mom had a very big…I think it’s also a spiritual pain. She asked me a very shocking question, “Am I a bad mom?” but to me this is a very obvious spiritual pain. She’d feel like her role, her problems, her decisions, what she’s done may have hurt her child, or given him more pain.”

Another theme of spiritual distress was difficulty in communication at the end of life, specifically how the provider felt that death was a sensitive subject that patients and families did not want to approach.

“…whether or not the friends and family around him can accept his emotions, how they interact, or if the communication between family members is minimal or not effective, his emotions may not have a way out.”

### Challenges to providing spiritual care

Participants listed the relative emotional, physical, and social status of the patient, especially altered consciousness, as one of the greatest challenges to effectively providing spiritual care for their patients.

“And for some patients who are referred later, we predict that these patients will have some things that they struggle to let go of, but his state of consciousness may also make it difficult for us to assess and help them in these aspects.”

An emphatically and frequently cited challenge to providing spiritual care was providers’ lack of preparedness to deliver spiritual care. This was expressed as unpreparedness in approaching the patient with spiritual concerns and difficulty identifying what constituted spiritual care and how to move forward with spiritual concerns when they were expressed to the provider.

“…I can’t define it. To me I won’t know if this is called spiritual care.”

“….because there isn’t an easier way to let me know what I’m doing is spiritual are, then if there are professionals helping together that’d be better, because this is still too intangible.”

## Discussion

The objective of this study was to gauge Taiwanese hospice physician and nurse perceptions of spiritual dimensions of end of life care from their own experiences and practices working in an academic tertiary inpatient palliative care unit with Buddhist origins. While literature on hospice care in Taiwan has made mention of spiritual themes at the end of life, to our knowledge, this is one of the first studies which focuses on the topic of spiritual care at the end of life from Taiwanese hospice physician and nurse perspectives. We developed a preliminary understanding of Taiwanese spiritual care in hospice care from the perspective of physicians and nurses through a directed content analysis of semi-structured interviews. Content was organized by three broad categories: definitions of spiritual care, sources of spiritual distress, and challenges in providing spiritual care.

Regarding how providers define spiritual care, we found that our participants frequently noted that spiritual care was difficult to provide a concrete definition for. Providers then came up with definitions of spiritual care focused on outlining patient beliefs and values, providing presence, and addressing physical symptoms as well as emotional needs. Intermittent but inconsistent reference was given to God or a higher power. Our participants also noted that sources of spiritual distress included the afterlife, burdening one’s family, and roadblocks in communication. Providers named the social unacceptability of candid discussions as playing a role in their workflow regarding spiritual care. Lastly, in response to being asked about challenges to spiritual care, our participants reported the general state of the patient and providers’ lack of preparedness to be barriers to provision of spiritual care. A lack of time and lack of professional training in spiritual care are sentiments that have previously been echoed in numerous Western hospice care settings by healthcare staff [21–23].

### Clinical implications

Despite institutional leanings toward promotion of spiritual care in the NTUH hospice through its Buddhist origins, physicians and nurses report definitions of spiritual care that focus on broader patient needs and do not necessarily present provision of spiritual care as a discrete phenomenon. The present study highlights that providers believe spiritual care to address basic goals in improving patient quality of life during serious illness but offer differing definitions of the scope of spiritual care practice in healthcare. Combined with providers’ self-perceived lack of preparedness to address spiritual concerns, this paucity of consistent, discrete definitions of spiritual care may stem from both a lack of professional training in spiritual care and a lack of clear definitions and roles for spiritual care on a more global scale.

As in other East Asian countries, Taiwanese hospice care arose largely from adopting Western concepts while retaining many of its own sociocultural roots [1, 24]. Studies in Taiwanese inpatient settings have defined spiritual care with elements of existentialism, religiosity, and personalized care, with documentation in the literature of spiritual care curricula developed specifically for nursing staff in Taiwan using such elements; presence of such curricula may vary greatly from institution to institution [1, 25–27]. In our study, we noted frequent mentions of interpersonal connectedness and fear of repercussions of not treating family well in the afterlife; physicians and nurses described that patient spiritual distress was intertwined with how much the patient felt they contributed to their families in life. The issue of such social merit, or lack thereof, causing spiritual distress at the end of life may represent a potential avenue for intervention in future hospice and spiritual care practice. Other East Asian contexts such as Japanese end of life literature have defined spiritual care as the act of addressing a lack of meaning and spiritual distress, as well as the loss of relationships, worth, and sense of having a future [24, 28]. This emphasis on relationship loss as a key cause of spiritual distress parallels a qualitative study in the Midwestern United States which interviewed dying cancer patients about their experiences of illness; however, unlike in this American study, our hospice healthcare staff did not consistently mention relationships to God as an essential part of spiritual care [29]. Our results from our population of mostly non-religious healthcare providers are surprisingly compatible with a study surveying Jordanian Muslim nurses, whom despite their overt religious identification reported providing spiritual care that was more existential rather than religious in nature and infrequently made explicit references to God [30].

In relation to the issue of communication at the end of life, hospice physicians and nurses made mention of difficulties discussing general end of life concerns openly, despite the fact that we did not ask nor prompt providers about sociocultural death taboos known previously in literature [16, 31, 32]. A survey randomly sampling clinic patients and their relatives in Taiwan revealed that most patients reported valuing autonomy with respect to a diagnosis of cancer, whereas relatives were more likely to value the principle of beneficence; this indicates that the influence of truth-telling in Chinese culture may differ based on one’s position as the patient or family, and candid conversations on end of life issues may be limited more by family factors rather than a patient’s desire to not know the seriousness of their disease [32]. Family-oriented decision making has been noted to influence DNR signing among elderly patients in Hong Kong, another ethnically Chinese setting [31]. While the emphasis which providers placed on communication and lack of candid discussion indicates that truth-telling may influence spiritual care provision, providers did not specify whether challenges arose from talking to patient or to family; rather, they referenced the difficulty of having conversations about end of life care in general. This is consistent with previous Taiwanese literature noting logistical difficulties in provision of patient-centered care by healthcare staff due to such communication concerns [15–18]. As a lack of candid discussion of terminal illness has been associated with lower respect for autonomy and decision-making participation among elderly Taiwanese patients, further investigation is warranted to determine an ideal balance between respect for patient autonomy and respect for familial sociocultural norms [14]. Potential future work includes developing family psychosocial interventions to reconcile “truth-telling” of serious illness with traditional practices that obscure adverse health circumstances, as well as training healthcare staff to approach candid conversations about end of life issues such as spiritual distress in a culturally-sensitive manner [14]. Further investigation is also warranted to determine if barriers to spiritual care stem from difficulty in discussing spiritual distress in particular or difficulty discussing any subject related to death in general.

### Study limitations

There are several limitations to this investigation. First, it is a single-institution study encompassing a largely non-religious sample of physicians and nurses. Findings in this study reflect a subculture of inpatient practice in Taiwan and are not necessarily applicable to other hospice practices, such as in Central or Southern Taiwan. Of note, most of our participants did not personally identify as spiritual or religious despite working in a hospice with a Buddhist background. This may have impacted the perspectives they were willing to share about spiritual care, as well as their interpretation of patients’ spiritual needs in the inpatient setting. Second, although participant information was protected and anonymous, a positive bias toward wanting to provide spiritual care may be influenced by the formal professional expectation to provide spiritual care within the hospice itself. Third, despite our efforts to recruit as many of our staff as possible for a comprehensive view of how spiritual care is practiced in our hospice, physicians and nurses spending more time near the inpatient palliative care unit were more readily available for interviews.

Additionally, limitations in our analysis include influence from summative or quantitative paradigms as the number of times participants volunteered themes influenced our perceived importance of said themes; while this method promoted fair representation of participant perspectives, it also came at the expense of presenting more rich and thick interview content as described in other qualitative studies [19]. Lastly, we did not separate perspectives of physicians from nurses during content analysis but treated them as a whole. Despite more extensive literature on initiatives in spiritual competency for nurses than physicians in Taiwan, the ramifications of these training differences for physician and nurse perspectives on spiritual care did not stand out in the present study and this represents another potential limitation [8, 11, 13, 26]. More research on processes of education and training in spiritual care in Taiwan is needed, especially for physicians, to elucidate any key differences between physicians and nurses in spiritual care practice.

## Conclusions

Using a qualitative methodological approach, we identified several themes from Taiwanese physicians and nurses’ self-reported practices of spiritual care in a single academic institution’s palliative care unit. Our study identified a key sample of physician and nurses working in a longstanding hospice ward with a Buddhist background. Many of these participants reported spiritual care to be difficult to define in practice. To provide a definition of spiritual care, providers responded with understanding patient values and beliefs to assist with coping, providing presence for patients, and relating spiritual care to physical and psychological phenomena. Themes in spiritual distress among patients and families involved apprehension over the afterlife, broken relationships with family, and difficulty attaining candid discussions regarding one’s situation in the end of life setting. Challenges to providing spiritual care included the state of the patient, such as their consciousness, and lack of confidence, time, and formal preparedness in delivering spiritual care.

## Data Availability

The data that support the findings of this study are available on request from the corresponding author. The data are not publicly available due to privacy and ethical restrictions.

## Abbreviations

NTUH: National Taiwan University Hospital
CBC: Clinical Buddhist Chaplain
